# Degradation of EGFR on lung epithelial cells by neutrophil elastase contributes to the aggravation of pneumococcal pneumonia

**DOI:** 10.1016/j.jbc.2023.104760

**Published:** 2023-04-27

**Authors:** Toshihito Isono, Satoru Hirayama, Hisanori Domon, Tomoki Maekawa, Hikaru Tamura, Takumi Hiyoshi, Kridtapat Sirisereephap, Shoji Takenaka, Yuichiro Noiri, Yutaka Terao

**Affiliations:** 1 Division of Microbiology and Infectious Diseases, Graduate School of Medical and Dental Sciences, Niigata University, Niigata, Japan; 2 Center for Advanced Oral Science, Graduate School of Medical and Dental Sciences, Niigata University, Niigata, Japan; 3 Division of Periodontology, Graduate School of Medical and Dental Sciences, Niigata University, Niigata, Japan; 4 Faculty of Dentistry, Chulalongkorn University, Bangkok, Thailand; 5 Division of Cariology, Operative Dentistry and Endodontics, Department of Oral Health Science, Niigata University Graduate School of Medical and Dental Sciences, Niigata, Japan

**Keywords:** epidermal growth factor receptor, epidermal growth factor, Akt, neutrophil, epithelial cell, cell proliferation

## Abstract

Pneumococcus is the main cause of bacterial pneumonia. Pneumococcal infection has been shown to cause elastase, an intracellular host defense factor, to leak from neutrophils. However, when neutrophil elastase (NE) leaks extracellularly, it can degrade host cell surface proteins such as epidermal growth factor receptor (EGFR) and potentially disrupt the alveolar epithelial barrier. In this study, we hypothesized that NE degrades the extracellular domain (ECD) of EGFR in alveolar epithelial cells and inhibits alveolar epithelial repair. Using SDS-PAGE, we showed that NE degraded the recombinant EGFR ECD and its ligand epidermal growth factor, and that the degradation of these proteins was counteracted by NE inhibitors. Furthermore, we confirmed the degradation by NE of EGFR expressed in alveolar epithelial cells *in vitro*. We showed that intracellular uptake of epidermal growth factor and EGFR signaling was downregulated in alveolar epithelial cells exposed to NE and found that cell proliferation was inhibited in these cells These negative effects of NE on cell proliferation were abolished by NE inhibitors. Finally, we confirmed the degradation of EGFR by NE *in vivo*. Fragments of EGFR ECD were detected in bronchoalveolar lavage fluid from pneumococcal pneumonia mice, and the percentage of cells positive for a cell proliferation marker Ki67 in lung tissue was reduced. In contrast, administration of an NE inhibitor decreased EGFR fragments in bronchoalveolar lavage fluid and increased the percentage of Ki67-positive cells. These findings suggest that degradation of EGFR by NE could inhibit the repair of alveolar epithelium and cause severe pneumonia.

Community-acquired pneumonia (CAP) has high morbidity and mortality rates worldwide (1), and severe CAP can cause sepsis and subsequent acute respiratory distress syndrome (2). *Streptococcus pneumoniae* is the primary causative microorganism of CAP (3). The main treatment for CAP is antimicrobial therapy; however, it has been severely hampered by the increase in antimicrobial-resistant strains of *S. pneumoniae* (4). Current pneumococcal vaccines have limited efficacies against specific serotypes. Owing to the diversity of pneumococcal serotypes and serotype replacement, CAP cannot be completely prevented (5). In addition, it is not highly effective in protecting against pneumococcal infection, since the antigens are not proteins but polysaccharides (6). Therefore, elucidating the mechanisms of pathogenesis and severity of pneumococcal disease and developing alternative treatment and prevention methods for antimicrobial agents and current vaccines should be emphasized.

Pneumococcal pneumonia is characterized by an excessive infiltration of neutrophils into the lung tissue (7). Neutrophils eliminate pathogens; however, *S. pneumoniae* is not killed by neutrophils. *S. pneumoniae* lyses neutrophils with its toxin pneumolysin and leaks elastase from neutrophils (8). Neutrophil elastase (NE) degrades constituent proteins of lung tissue, such as elastin (9), collagen (9), and E-cadherin (10), and acts as an exacerbating factor in acute and chronic pulmonary diseases, including acute respiratory distress syndrome (11), cystic fibrosis (12), and chronic obstructive pulmonary disease (13). Loss of epithelial cell integrity and shedding are common in these diseases, including pneumococcal pneumonia; however, the mechanisms of epithelial destruction by NE are not completely understood. Since NE degrades receptor proteins, such as the C5a receptor (14) and Toll-like receptor (15), expressed on the plasma membrane, we hypothesized that NE might also degrade epidermal growth factor receptor (EGFR) that is expressed in the alveolar epithelium and is involved in maintaining epithelial tissue.

EGFR is a transmembrane protein with a molecular weight of 170 kDa and is responsible for mammalian epithelial maintenance; it consists of one each of extracellular domain (ECD), transmembrane domain, and intracellular domain (16). Binding of epidermal growth factor (EGF) to the EGFR results in receptor dimerization, autophosphorylation of two domains of cytoplasmic tyrosine kinase of the receptor, and subsequent induction of cell proliferation (17). Aberrations in EGFR signaling are associated with respiratory diseases, such as pulmonary fibrosis (18), cancer (19), and asthma (20). Therefore, abnormal EGFR signaling might be involved in exacerbating pneumococcal pneumonia.

In this study, we examined whether NE degrades EGFR expressed in cells, using alveolar epithelial cells and a mouse model of pneumococcal pneumonia.

## Results

### NE degrades the ECD of EGFR and EGF

**Figure 1:**
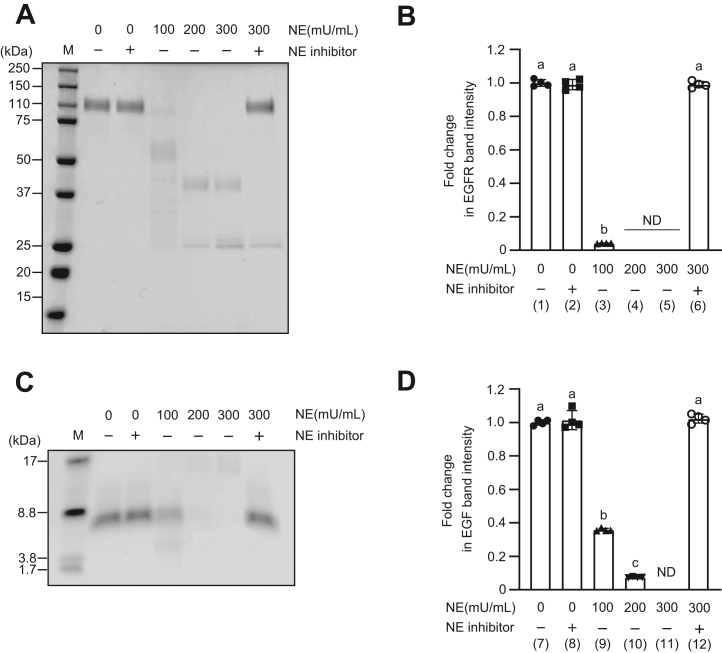
**Neutrophil elastase (NE) degrades recombinant epidermal growth factor receptor (rEGFR) extracellular domain (ECD) and recombinant epidermal growth factor (rEGF).***A* and *C*, rEGFR ECD and rEGF were exposed to NE (100–300 mU/ml) in the presence or the absence of NE inhibitor for 3 h at 37 °C. Proteins were separated by SDS-PAGE followed by Coomassie brilliant blue staining. The representative gel images are shown. M, molecular weight marker. *B* and *D*, quantification of intensities of EGFR and EGF bands. Data represent mean ± SD of four individual experiments and were evaluated using ANOVA with Tukey’s multiple comparison test. Unless otherwise indicated, no significant differences were observed between groups. Differences in letters between bars (a, b) indicate statistically significant differences between groups. Unless indicated, no significant differences were observed between groups (*p* < 0.05). (1) *versus* (2): *p* = 0.9623, (1) *versus* (3): *p* < 0.0001, (1) *versus* (6): *p* = 0.9712, (2) *versus* (3): *p* < 0.0001, (2) *versus* (6): *p* > 0.9999, (3) *versus* (6): *p* < 0.0001, (7) *versus* (8): *p* = 0.9734, (7) *versus* (9): *p* < 0.0001, (7) *versus* (10): *p* < 0.0001, (7) *versus* (12): 0.9975, (8) *versus* (9): *p* < 0.0001, (8) *versus* (12): *p* < 0.0001, (9) *versus* (10): *p* < 0.0001, (9) *versus* (12): *p* < 0.0001, (10) *versus* (12): *p* < 0.0001. ND, not detected.

EGFR exposes its ECD on the plasma membrane. We hypothesized that NE leaked from neutrophils targets the ECD of EGFR and degrades it. Therefore, we analyzed whether NE degraded the ECD of recombinant EGFR (rEGFR ECD). rEGFR ECD was exposed to NE (100–300 mU/ml) in the presence or the absence of NE inhibitor for 3 h and separated using SDS-PAGE to quantify the intensity of EGFR bands (Figs. 1, *A* and *B* and S1*A*). The intensity of EGFR bands significantly decreased in a dose-dependent manner with the addition of NE, whereas significantly higher band intensity of EGFR was detected in EGFR treated with NE inhibitor along with NE (300 mU/ml) (Fig. 1*B*). We also analyzed whether NE also degrades EGF, one of the ligands for EGFR. Recombinant EGF was exposed to NE (100–300 mU/ml) for 3 h in the presence or the absence of NE inhibitors and separated by SDS-PAGE to quantify the intensity of EGF bands (Figs. 1, *C* and *D* and S1*B*). The band intensity of EGF was significantly decreased in a dose-dependent manner with the addition of NE, whereas significantly higher band intensity of EGF was detected in EGF treated with NE inhibitor along with NE (300 mU/ml). These results suggest that NE degrades both EGFR ECD and EGF.

### NE degrades EGFR expressed on the surface of alveolar epithelial cells

**Figure 2:**
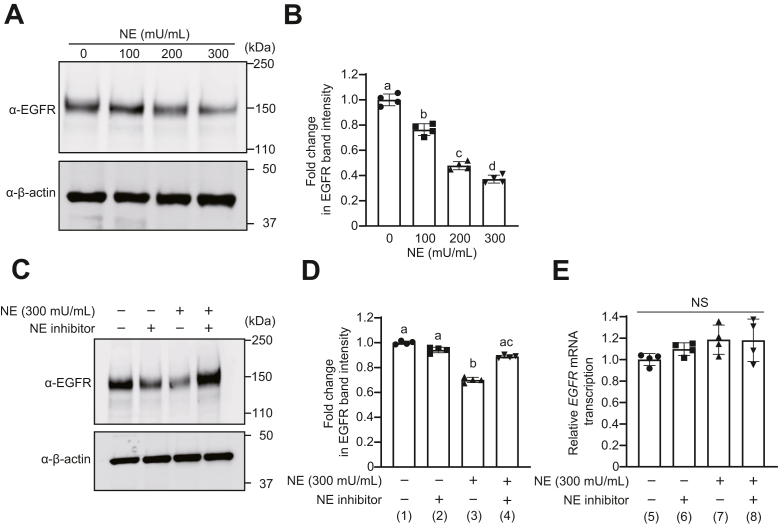
**Neutrophil elastase (NE) degrades epidermal growth factor receptor (EGFR) expressed on A549 cells.** A549 cells were exposed to NE (100–300 mU/ml) for 20 min. *A*, EGFR expression was determined by Western blotting. *B*, quantification of EGFR band intensity. A549 cells were exposed to NE (300 mU/ml) in the presence or the absence of NE inhibitor (100 μg/ml). *C*, EGFR expression was determined by Western blotting. *D*, quantification of EGFR band intensity. *E*, real-time PCR was performed to analyze the mRNA level of EGFR. The relative quantity of EGFR mRNA was normalized to that of GAPDH mRNA. Data represent mean ± SD of four individual experiments and were evaluated using ANOVA with Tukey’s multiple comparison test. Unless otherwise indicated, no significant differences were observed between groups. Differences in letters between bars (a, b) indicate statistically significant differences between groups (*p* < 0.05). 0 *versus* 100: *p* < 0.0001, 0 *versus* 200: *p* < 0.0001, 0 *versus* 300: *p* < 0.0001, 100 *versus* 200: *p* < 0.0001, 100 *versus* 300: *p* < 0.0001, 200 *versus* 300: *p* < 0.0001, (1) *versus* (2): *p* = 0.1428, (1) *versus* (3): *p* < 0.0001, (1) *versus* (4): *p* < 0.1383, (2) *versus* (3): *p* < 0.0001, (2) *versus* (4): *p* < 0.0001, (3) *versus* (4): *p* < 0.0001, (5) *versus* (6): *p* = 0.7067, (5) *versus* (7): *p* = 0.2178, (5) *versus* (8): *p* = 0.2406, (6) *versus* (7): *p* = 0.7593, (6) *versus* (8): *p* = 0.7949, (7) *versus* (8): *p* = 0.9999. NS, not significant.

Whether EGFR expressed in the alveolar epithelial cell line A549 was also degraded was analyzed *in vitro*. After NE treatment of A549 cells, total proteins were extracted, and EGFR was detected in cell extracts by Western blotting. The band intensity of EGFR significantly reduced in a dose-dependent manner by the addition of NE (Fig. 2, *A* and *B* and S2*A*). Significantly higher band intensity of EGFR was detected in A549 cells treated with NE inhibitor along with NE (300 mU/ml) than in cells treated with NE alone (Fig. 2, *C* and *D* and S2*B*). To confirm that the decrease in EGFR in NE-treated cells was not because of a decreased mRNA expression, mRNA was extracted from the cells, and real-time PCR was performed (Fig. 2*E*). No significant difference in EGFR mRNA levels was observed between NE-treated and untreated A549 cells. These results indicated that NE degraded EGFR expression in living cells without affecting the mRNA level of EGFR.

### Degradation of EGFR by NE results in a decreased EGF binding to EGFR

**Figure 3:**
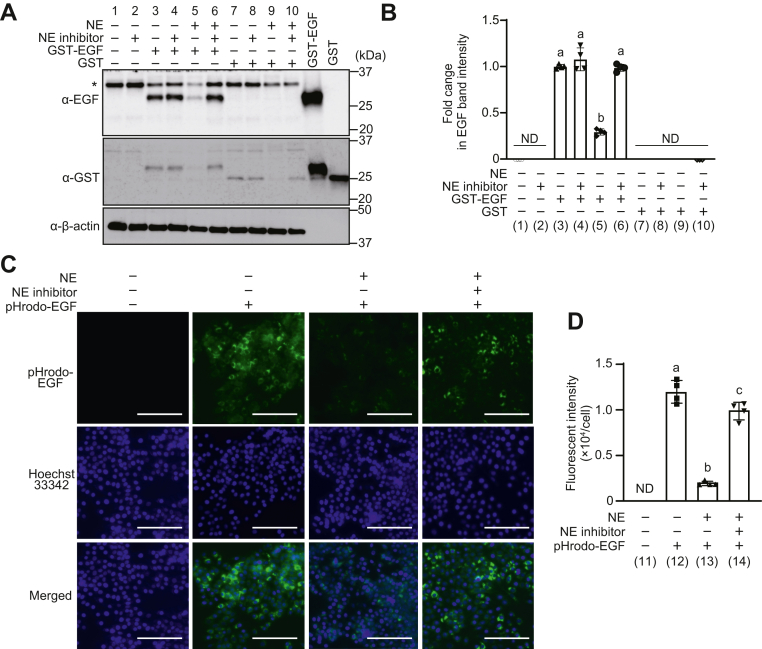
**Neutrophil elastase (NE) exposure decreases epidermal growth factor (EGF) uptake in A549 cells.** A549 cells were exposed to NE (300 mU/ml) in the presence or the absence of NE inhibitor (100 mg/ml) for 20 min. *A*, cells were stimulated with glutathione-*S*-transferase (GST)-EGF or GST for 10 min. GST-EGF in the cell lysate was detected by Western blotting using anti-EGF and anti-GST antibodies. ∗EGF precursor. *B*, quantification of EGF band intensity. *C*, cells exposed to NE were treated with pH-rodo EGF for 10 min, and internalized pH-rodo EGF was detected without fixation. Representative images of cells are presented. pHrodo-EGF, *green*; Hoechst 33342, *blue*; magnification, 20×; scale bar represents 100 μm. *D*, quantification of fluorescence intensity of pH-rodo EGF. Data represent mean ± SD of four individual experiments and were evaluated using ANOVA with Tukey’s multiple comparison tests. Unless otherwise indicated, no significant differences were observed between groups. Differences in letters between bars (a, b) indicate statistically significant differences between groups (*p* < 0.05). (3) *versus* (4): *p* = 0.9571, (3) *versus* (5): *p* < 0.0001, (3) *versus* (6): *p* > 0.9999, (4) *versus* (5): *p* < 0.0001, (4) *versus* (6): *p* > 0.9999, (5) *versus* (6): *p* < 0.0001, (12) *versus* (13): *p* = 0.0042, (12) *versus* (14): *p* = 0.0585, (13) *versus* (14): *p* = 0.0090. ND, not detected.

**Figure 4:**
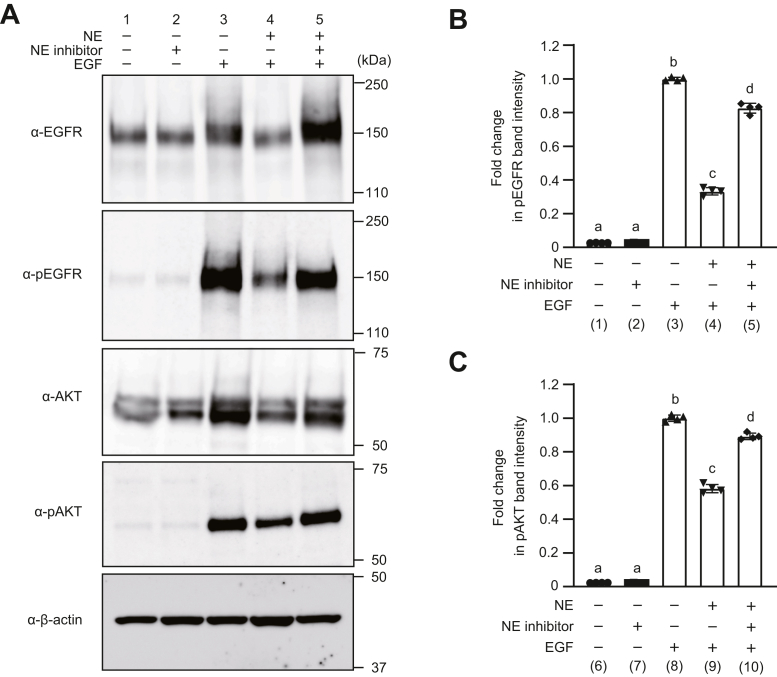
**Neutrophil elastase (NE) exposure downregulates the phosphorylation of epidermal growth factor receptor (EGFR) and AKT.** A549 cells were exposed to NE (300 mU/ml) in the presence or the absence of NE inhibitor (100 mg/ml) for 20 min. The culture medium was replaced with fresh Dulbecco’s modified Eagle's medium (DMEM), and cells were stimulated with epidermal growth factor (EGF) (50 ng/ml). *A*, expression of EGFR, phosphorylated EGFR (pEGFR), AKT, and phosphorylated AKT (pAKT) was determined by Western blotting. Representative Western blotting images are shown. *B, C*, quantification of band intensities of pEGFR and pAKT. Data represent mean ± SD of four individual experiments and were evaluated using ANOVA with Tukey’s multiple comparison tests. Unless otherwise indicated, no significant differences were observed between groups. Differences in letters between bars (a, b) indicate statistically significant differences between groups (*p* < 0.05). (1) *versus* (2): *p* > 0.9999, (1) *versus* (3): *p* < 0.0001, (1) *versus* (4): *p* < 0.0001, (1) *versus* (5): *p* < 0.0001, (2) *versus* (3): *p* < 0.0001, (2) *versus* (4): *p* < 0.0001, (2) *versus* (5): *p* < 0.0001, (3) *versus* (4): *p* < 0.0001, (3) *versus* (5): *p* < 0.0001, (4) *versus* (5): *p* < 0.0001, (6) *versus* (7): *p* > 0.9999, (6) *versus* (8): *p* < 0.0001, (6) *versus* (9): *p* < 0.0001, (6) *versus* (10): *p* < 0.0001, (7) *versus* (8): *p* < 0.0001, (7) *versus* (9): *p* < 0.0001, (7) *versus* (10): *p* < 0.0001, (8) *versus* (9): *p* < 0.0001, (8) *versus* (10): *p* < 0.0001, and (9) *versus* (10): *p* < 0.0001.

When EGF binds to the ECD of EGFR, the EGF–EGFR complex is internalized into the cell for subsequent signaling (21). The effect of EGFR degradation by NE on EGF internalization into the cell was analyzed using glutathione-*S*-transferase (GST)-fused EGF (GST-EGF). First, to confirm that the GST tag itself did not activate EGFR, A549 cells were stimulated with EGF, GST-EGF, or GST, and phosphorylated EGFR (pEGFR) was detected by Western blotting (Fig. S3*A*). The band intensity of pEGFR was similar in GST-stimulated and untreated cells but increased in cells stimulated with EGF or GST-EGF. Next, A549 cells exposed to NE in the presence or the absence of NE inhibitors were stimulated with GST-EGF. The uptake of GST-EGF by A549 cells was detected by Western blotting of A549 cell lysates using anti-EGF and anti-GST antibodies (Fig. 3*A*). The intensity of bands specific to anti-EGF antibody was reduced in NE-treated cells compared with that in untreated cells (lanes 3 and 5 in Figs 3, *A* and *B* and S3*B*). However, in cells treated with NE and NE inhibitors, bands specific to GST-EGF were detected at significantly higher intensities than those in cells treated with NE (lanes 5 and 6 in Figs 3, *A* and *B* and S3, *B* and *C*). Western blotting images of A549 cell lysate with anti-EGF antibody showed a band of approximately 37 kDa in addition to GST-EGF. EGF has been reported to have a precursor of 20 to 165 kDa in a study using mouse kidney (22). Since A549 cells also express EGF (23), it is possible that cell-derived precursors were detected with anti-EGF antibody. The intensity of the band of approximately 37 kDa was decreased in NE-treated cells compared with untreated A549 cells. In contrast, the intensity increased in cells treated with NE and NE inhibitors. Figure 1, *B* and *C* demonstrates that NE degrades EGF and that EGF precursors expressed in A549 cells can be degraded by NE. In addition, internalization of EGF bound to EGFR into A549 cells was visualized using pHrodo-EGF (24). A549 cells treated with NE in the presence or the absence of NE inhibitor were incubated with pHrodo-EGF, and fluorescence of internalized pHrodo-EGF was microscopically observed and quantified. The fluorescence intensity of pHrodo-EGF was significantly lower in NE-treated cells than in untreated cells. However, consistent with the results in Figure 4, *A* and *B*, the fluorescence intensity was significantly higher in cells treated with NE and NE inhibitors than in cells treated with NE alone (Fig. 3, *C* and *D*). Therefore, when EGFR expressed on the surface of A549 cells is degraded by NE, the amount of EGF bound to and internalized by EGFR is reduced.

### Degradation of EGFR by NE results in attenuated activation of EGFR signaling

To analyze the effect of EGFR degradation by NE on EGF–EGFR signaling, we analyzed the activation of EGFR and AKT that is involved in essential downstream pathways of EGFR signaling (25). NE-treated A549 cells were stimulated by EGF, and pEGFR and phosphorylated AKT (pAKT) levels in cell lysates were detected by Western blotting (Fig. 4*A*). Upon EGF stimulation, the band intensities of pEGFR and pAKT were significantly lower in NE-treated cells than in untreated cells (lanes 3 and 4 in Figs. 4, *A*–*C* and S4). However, significantly higher band intensities of pEGFR and pAKT were noticed in cells cotreated with NE and NE inhibitor than in NE-treated cells (lanes 4 and 5 in Figs. 4, *A*–*C* and S4). These results indicate that EGFR degradation by NE hampers the activation of EGFR signaling induced by EGF.

### Degradation of EGFR by NE inhibits EGF-mediated proliferation of lung epithelial cells

**Figure 5:**
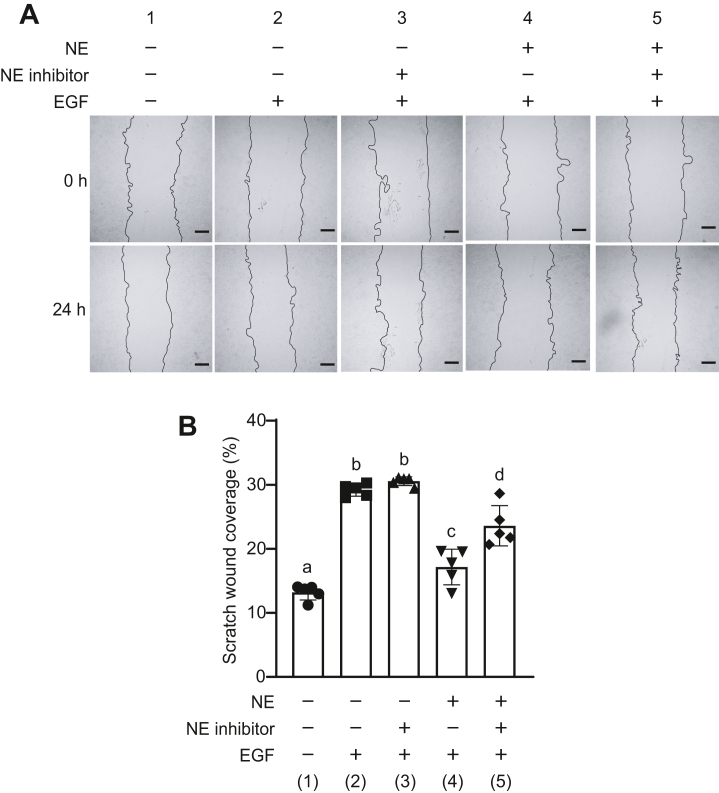
**Neutrophil elastase (NE) exposure inhibits wound closure induced by epidermal growth factor (EGF).** Monolayer of A549 cells was scratched with a pipette tip, and cells were exposed to NE (300 mU/ml) in the presence or the absence of NE inhibitor (100 mg/ml) for 20 min. The culture medium was replaced with fresh Dulbecco’s modified Eagle's medium (DMEM), and cells were cultured in complete medium supplemented with EGF (50 ng/ml) for 24 h. *A*, representative microscopic images immediately after NE exposure (0 h) and after 24 h are shown. Magnification, 4×; scale bar represents 100 μm. *B*, the relative wound healing area was calculated as the ratio of wound area reduced after 24 h to the initial wound area (0 h). Data represent mean ± SD of five individual experiments and were evaluated using ANOVA with Tukey’s multiple comparison tests. Unless otherwise indicated, no significant differences were observed between groups. Differences in letters between groups are statistically significant (*p* < 0.05). (1) *versus* (2): *p* < 0.0001, (1) *versus* (3): *p* < 0.0001, (1) *versus* (4): *p* = 0.04, (1) *versus* (5): *p* < 0.0001, (2) *versus* (3): *p* = 0.8093, (2) *versus* (4): *p* < 0.0001, (2) *versus* (5): *p* = 0.0024, (3) *versus* (4): *p* < 0.0001, (3) *versus* (5): *p* = 0.0002, and (4) *versus* (5): *p* = 0.0005.

Activation of EGFR by ligands, such as EGF, promotes cell proliferation and migration, thereby promoting the repair of damaged epithelium (26). We performed a wound healing assay to analyze whether the suppression of signaling owing to EGFR degradation by NE inhibited wound healing in lung epithelial tissues (Fig. 5, *A* and *B*). Because fetal bovine serum (FBS) present in the growth medium contains growth factors, we first confirmed that the addition of EGF further promoted cell proliferation. The rate of wound closure after stimulation with EGF for 24 h was quantified by image analysis. EGF-stimulated A549 cells showed a significantly higher rate of wound closure than did the unstimulated cells (lanes 1, 2, and 3 in Fig. 5, *A* and *B*). NE-treated cells stimulated with EGF for 24 h showed approximately 13% lower rates of wound closure than did non–NE-treated cells (lanes 2 and 4 in Fig. 5, *A* and *B*). In addition, the rate was significantly higher in NE-treated cells by approximately 8% than in cells treated with NE and NE inhibitors (lanes 4 and 5 in Fig. 5, *A* and *B*). These findings suggested that EGFR degradation by NE inhibited EGF-mediated cell proliferation.

### The EGFR ECD is detected in BALF of pneumococcus-infected mice

**Figure 6:**
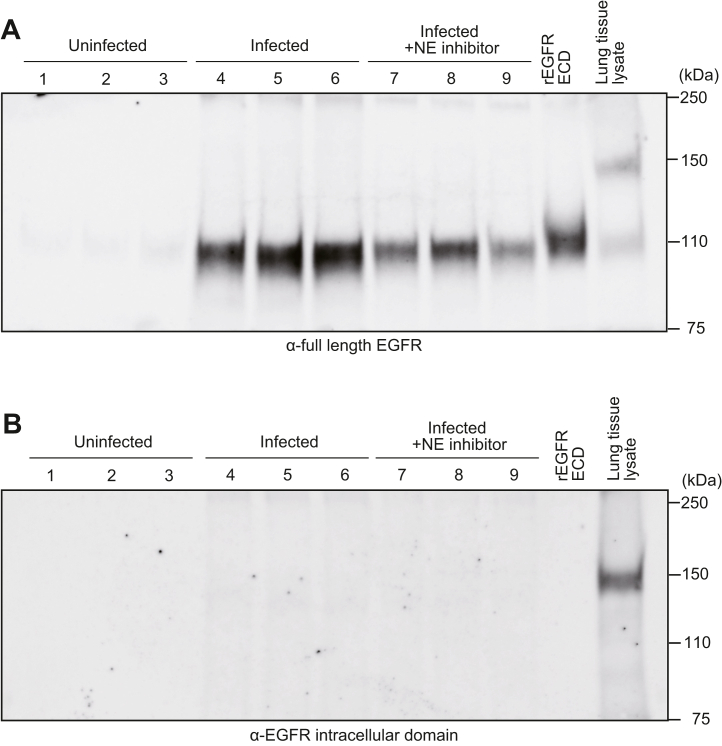
**Pneumococcal infection increases the amount of epidermal growth factor receptor (EGFR) fragments in the BALF of mice.** Mice (n = 6/group) were infected with pneumococcus and then intraperitoneally administered PBS (infected group: 4–6) or neutrophil elastase (NE) inhibitor (infected + NE inhibitor group: 7–9) immediately after infection and three times at every 6 h. Uninfected mice were injected with PBS only (uninfected group: 1–3). Western blotting was performed to detect EGFR in BALF using an antibody specific to full-length EGFR (*A*) or to the intracellular domain of EGFR (*B*). Recombinant EGFR (rEGFR) extracellular domain (ECD) and mouse lung lysate were included as controls. Representative Western blot images (three samples from each group) are shown.

We have shown that NE degrades EGFR on the surface of alveolar epithelial cells *in vitro*. Therefore, we analyzed whether degraded EGFR fragments are detected in the BALF of pneumococcus-infected mice and whether NE is involved in this process. After intratracheal administration of *S. pneumoniae*, NE inhibitor or vehicle control (PBS) was intraperitoneally administered thrice every 6 h immediately after infection. BALF samples were collected and subjected to Western blotting using an antibody against the full-length of EGFR (Figs. 6*A* and S5*A*). EGFR-specific bands were detected in the BALF of mice in the infected group but not in the uninfected group. The intensity of the EGFR bands in BALF was reduced in mice administered NE inhibitor after infection compared with the infected group. Clear bands were detected when the same antibody was used against rEGFR ECD and mouse lung tissue lysate used as control. Western blotting was performed on the same samples using an antibody that specifically recognizes the intracellular domain of EGFR (Figs. 6*B* and S6*B*). No bands were detected in either BALF sample. An obvious band was detected in mouse lung tissue lysate but not in rEGFR ECD. These results suggest that the EGFR ECD is degraded and released into the BALF of pneumococcus-infected mice and that NE may be involved in this process.

### Pneumococcal infection reduces EGFR and Ki67 expression in lung tissue

**Figure 7:**
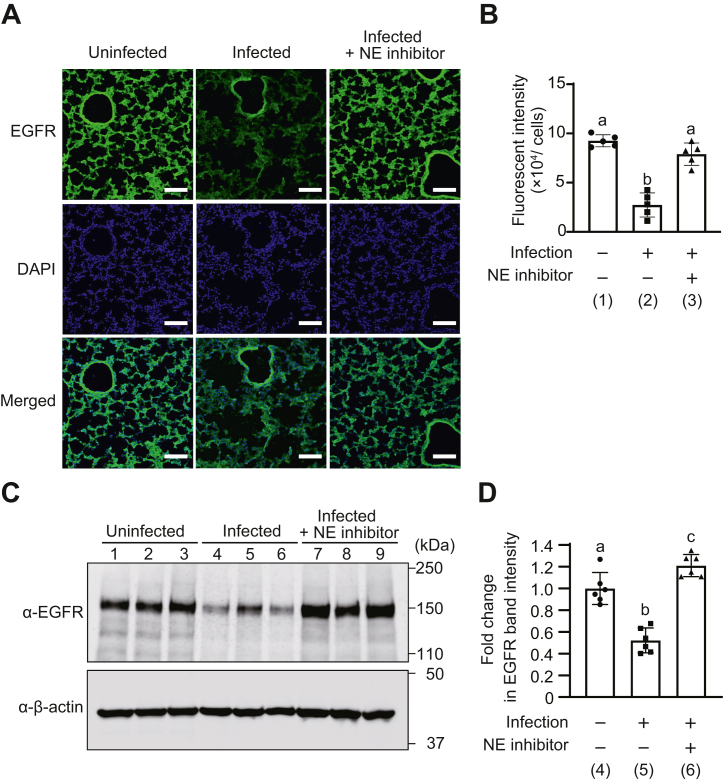
**Pneumococcal infection decreases epidermal growth factor receptor (EGFR) expression in mouse lung tissue.** Mice (n = 6/group) were infected with pneumococcus and then intraperitoneally administered PBS (infected group) or neutrophil elastase (NE) inhibitor (infected + NE inhibitor group) immediately after infection and three times at every 6 h. Uninfected mice were injected with PBS alone (uninfected group). *A*, representative fluorescence microscopic images of the lungs from mice stained with EGFR are shown. EGFR (*green*), 4′,6-diamidino-2-phenylindole (DAPI) (*blue*); magnification, 20×; scale bar represents 100 μm. *B*, quantification of fluorescence intensity of EGFR. *C*, EGFR expression in lung tissues was determined by Western blotting. Representative images of Western blot (three samples from each group) are shown. *D*, quantification of band intensity of EGFR. Data represent the mean ± SD and were evaluated using ANOVA with Tukey’s multiple comparison tests. Differences in letters between bars (a, b) indicate statistically significant differences between groups (*p* < 0.05). (1) *versus* (2): *p* < 0.0001, (1) *versus* (3): *p* = 0.1337, (2) *versus* (3): *p* < 0.0001, (4) *versus* (5): *p* < 0.0001, (4) *versus* (6): *p* = 0.0243, and (5) *versus* (6): *p* < 0.0001.

To confirm that EGFR degradation by NE occurs in lung tissue, we analyzed the amount of EGFR in lung tissues from model mice of pneumococcal pneumonia. Mice were infected with *S. pneumoniae* and treated with NE inhibitor or PBS, and lung tissue was harvested. Immunofluorescent staining was performed to detect EGFR expression in lung tissue (Fig. 7, *A* and *B*). EGFR expression reduced in infected mice treated with PBS compared with that in uninfected mice. In addition, EGFR expression increased in lung tissues of infected mice treated with NE inhibitor compared with that in infected mice treated with PBS. EGFR levels in lung tissues were detected by Western blotting also (Figs. 7, *C* and *D* and S6). Consistent with the immunofluorescence staining results, band intensities of EGFR reduced in the lung tissues of infected mice treated with PBS compared with those of uninfected mice. Furthermore, the band intensity of EGFR increased in the lung tissues of infected mice treated with NE inhibitor compared with that in mice treated with PBS. These results suggest that NE leakage by pneumococcal infection is involved in degrading EGFR on the surface of lung tissue cells.

**Figure 8:**
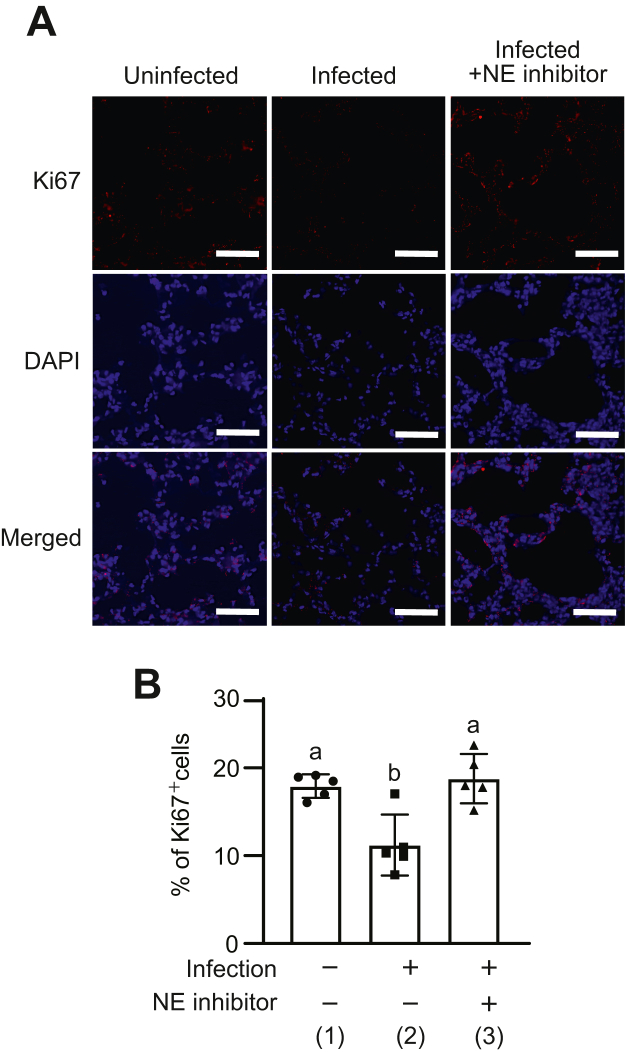
**Pneumococcal infection decreases Ki67 expression in lung tissue.***A*, representative fluorescence microscopic images of the lungs from mice stained with Ki67 are shown. Ki67 (*red*), 4′,6-diamidino-2-phenylindole (DAPI) (*blue*); magnification, 40×; scale bar represents 100 μm. *B*, data represent means ± SD and were evaluated using ANOVA with Tukey’s multiple comparison tests. Differences in letters between bars (a, b) indicate statistically significant differences between groups (*p* < 0.05). (1) *versus* (2): *p* = 0.0051, (1) *versus* (3): *p* = 0.8707, and (2) *versus* (3): *p* = 0.0021.

Since activation of EGFR promotes cell proliferation, it is predicted that a decrease in the amount of EGFR in lungs of mice infected with pneumococcus would result in a decrease in the expression of cell proliferation markers. Therefore, we finally detected Ki67, one of the cell proliferation markers in the lung tissue (27), by immunofluorescence staining and calculated the percentage of Ki67-positive cells (Fig. 8, *A* and *B*). The percentage of Ki67-positive cells in the infected mice treated with PBS was reduced compared with that in uninfected mice. In addition, the percentage of Ki67-positive cells in lung tissues of infected mice treated with NE inhibitor was increased compared with that of infected mice treated with PBS. These results suggest that degradation of EGFR by pneumococcal infection inhibits cell proliferation in lung tissue.

## Discussion

**Figure 9:**
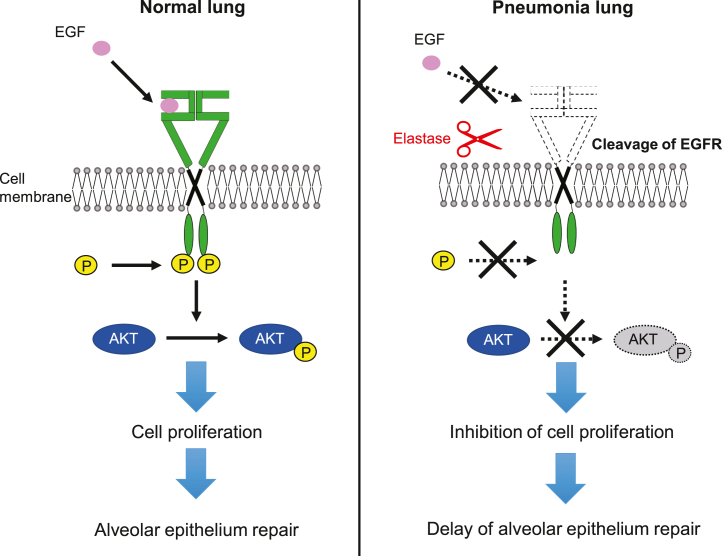
**Epidermal growth factor receptor (EGFR) cleavage by neutrophil elastase (NE) inhibits the repair of lung epithelial barrier.** NE degrades EGFR on epithelial cell membranes. Degradation of EGFR by NE decreases its binding to its ligand epidermal growth factor (EGF). Subsequently, AKT-mediated EGFR signaling of EGFR is downregulated, and alveolar epithelial growth is inhibited. Consequently, the repair of pulmonary epithelial barrier is inhibited.

Alveolar epithelial cells contribute to host defense by forming an epithelial barrier that provides physical defense against bacterial and viral stimuli and by producing surfactant proteins that promote phagocytosis and death of microbes (28). Loss of integrity of the epithelial barrier that is composed of alveolar epithelial cells is implicated in the development of pneumonia (29), and initiating tissue repair and restoring barrier function in response to epithelial damage are important. Therefore, we hypothesized that aberrations in molecules related to tissue repair contribute to the exacerbation of pneumococcal pneumonia and analyzed the interaction between EGFR and NE. We observed that NE degraded the ECD of EGFR. EGFR degradation by NE reduced the binding of EGF to EGFR and subsequently inhibited EGFR signaling and epithelial cell proliferation. These results suggest that EGFR degradation by NE leaked from neutrophils owing to pneumococcal infection can inhibit the repair of the epithelial barrier. A schematic diagram of the mechanism through which EGFR degradation by NE inhibits alveolar epithelial repair is shown in Figure 9. Previous studies have revealed the role of NE in pneumococcal pneumonia focusing on direct tissue destruction (30) and effects on immune cells (15, 31). Our findings deciphered a novel cause of severity of pneumonia, in which NE inhibits lung tissue repair.

NE degraded the EGFR ECD and produced multiple fragments from rEGFR ECD. Therefore, NE may possess multiple cleavage sites in EGFR as NE shows relatively broad elastase specificity (32). However, a single EGFR fragment was detected in the BALF of mice. Therefore, the cleavage activity of EGFR by NE may depend on the cleavage site.

Inflammatory cytokines, such as tumor necrosis factor (TNF) and interleukin (IL)-6, are released in large amounts in inflamed tissues of the lungs (33, 34). Interactions between these cytokines and EGFR signaling cause lung injury, and TNF inhibits TNF-induced excessive epithelial apoptosis by transactivating EGFR (35). However, inhibition of EGFR signaling by EGFR-tyrosine kinase inhibitors inhibits the transactivation of EGFR by TNF and promotes apoptosis (36). Cancer cells treated with EFGR-tyrosine kinase inhibitor show an increased expression of IL-6 mRNA (37, 38). IL-6 is increased in BALF of patients with acute respiratory distress syndrome (39), and high levels are associated with adverse events (40, 41). IL-6 may contribute to lung damage *via* direct action on the alveolar space (42). Therefore, we speculate that inhibition of EGFR signaling because of EGFR cleavage by NE inhibits alveolar epithelial regeneration and exacerbates lung injury caused by inflammatory cytokines released in pneumonia. However, no direct evidence has proved that EGFR degradation by NE exacerbates lung injury caused by TNF and IL-6, and further studies are needed.

In this study, sivelestat was used as NE inhibitor. Sivelestat has been used clinically to treat patients with acute lung injury associated with systemic inflammatory response syndrome (43). Inhibition of NE contributes to improved respiratory function in patients with respiratory disease, as shown in a multicenter clinical trial, in which, patients with acute lung injury treated with sivelestat sodium were weaned early from ventilators and had significantly increased survival rates (44, 45). In an animal model of pneumococcal pneumonia, sivelestat administration increases survival and decreases the number of bacteria in the blood (46, 47). In the present study, we demonstrated that sivelestat, an NE inhibitor, suppressed EGFR degradation. Therefore, sivelestat may contribute to the recovery of alveolar damage by NE, which is elevated in the lung tissue of pneumococcal pneumonia patients. Although sivelestat is not a standard clinical treatment for pneumococcal pneumonia (48), our results provide evidence that therapies using NE inhibitors, including sivelestat, are effective as adjunctive and antimicrobial therapies in pneumococcal pneumonia.

NE degraded not only EGFR but also its ligand EGF. In this study, we focused on the effect of NE on EGFR degradation on alveolar epithelial proliferation. The cells exposed to NE were stimulated with EGF after removal of NE, so the effect of NE on EGF degradation and cell proliferation was not analyzed. NE (300 mU/ml) completely degraded EGF. Our previous study showed that NE activity increased to an average of 431.6 mU/ml in BALF of pneumococcal-infected mice (15), suggesting that increased NE activity because of pneumococcal infection may degrade not only EGFR but also EGF and inhibit alveolar epithelial repair.

In summary, we demonstrated a novel mechanism of severe pneumococcal pneumonia, in which NE degrades EGFR expressed on alveolar epithelial cells and inhibits alveolar epithelial repair. In addition, our results suggest that NE inhibitors promote the recovery of alveolar epithelium damaged by pneumonia by inhibiting EGFR degradation by NE.

## Experimental procedures

### Bacterial and cell culture

Alveolar epithelial A549 cells (American Type Culture Collection CCL-185; RIKEN Cell Bank) were cultured in Dulbecco’s modified Eagle's medium (DMEM) supplemented with 10% FBS, 100 U/ml penicillin, and 100 μg/ml streptomycin (FUJIFILM Wako Pure Chemical) at 37 °C under 5% CO_2_.

*S. pneumoniae* strain D39 (serotype 2) was grown in trypticase soy broth (Becton Dickinson) at 37 °C.

### Cleavage of EGF and EGFR by NE

Human EGFR ECD (500 ng; R&D Systems) and human EGF (500 ng; R&D Systems) were treated with multiple doses of NE (100–300 mU/ml) in the presence or the absence of NE inhibitor, sivelestat sodium hydrate (100 μg/ml; ONO Pharmaceutical Co) at 37 °C for 3 h. Samples were separated by Tris–glycine–SDS-PAGE using 12% polyacrylamide gels (Bio-Rad) or tricine–SDS-PAGE with a 16% Peptide-PAGE mini (TEFCO). The gels were stained with Coomassie Brilliant Blue (Apro Science).

### NE stimulation of lung epithelial cells

A549 cells were treated with different doses (100, 200, and 300 mU/ml) of NE in the presence or the absence of NE inhibitor (100 μg/ml) for 20 min at 37 °C, and EGFR expression was analyzed by Western blotting using anti-EGFR antibody (catalog no.: ab52894; Abcam). Real-time PCR was performed as previously described, with some modifications (49). Total RNA was extracted from A549 cells using TRI reagent (Molecular Research Center). Complementary DNA was prepared from total RNA using a SuperScript VILO Master Mix (Thermo Fisher Scientific), and quantitative real-time PCR was performed using a StepOnePlus Real-time PCR system (Thermo Fisher Scientific) following the manufacturer's protocol. TaqMan probes (Thermo Fisher Scientific) were used to study the expression of *GAPDH* and *EGFR*. mRNA expression was normalized with respect to *GAPDH* expression and were calculated as fold changes relative to mRNA level of the control group.

### Construction of GST fused with EGF

GST-fused EGF (GST-EGF) was constructed as previously described with some modifications (50). Human EGF DNA (accession: NP_001954) was synthesized by Eurofins Genomics. The ORF of EGF was amplified from the synthetic DNA using primers 5′-GGGGGATCCGGGATAAATGATAAAATGGAA-3′ and 5′-CCCGAATTCTTAACATCCTATAGAACCTAC-3′ and then inserted into the BamHI–XmaI site of pGEX6P-1 vector (Cytiva). *Escherichia coli* strain Rosetta-gami B(DE3) was transformed with this plasmid. Transformants were incubated at 20 °C for 24 h in the presence of 100 μM isopropyl-β-D-thiogalactopyranoside (FUJIFILM Wako Pure Chemical). GST-EGF expressed in soluble fractions was purified using glutathione-Sepharose 4B beads (Cytiva), and purified GST-EGF was dialyzed against PBS. This genetic recombination experiment was approved by the safety committee for genetic modification experiments of Niigata University (no.: SD01711).

### Binding of EGF and EGFR

A549 cells were incubated with NE (300 mU/ml) in serum-free DMEM for 20 min at 37 °C in the presence or the absence of NE inhibitor (100 μg/ml); medium was replaced; and cells were stimulated with GST-EGF (500 ng/ml) for 10 min. Total protein was extracted, and internalized GST-EGF was detected by Western blotting using anti-EGF antibody (catalog no.: ab184265; Abcam) and anti-GST antibody (catalog no.: 2622; Cell Signaling Technology).

pHrodo-conjugated EGF (pHrodo-EGF; Thermo Fisher Scientific) was used to visualize the binding of EGF to EGFR, according to the manufacturer’s instructions. Briefly, A549 cells were treated with NE (300 mU/ml) in the presence or the absence of NE inhibitor (100 μg/ml) for 20 min and then kept on ice for 10 min. Cells were incubated in Live Imaging Solution (Thermo Fisher Scientific) containing pHrodo-EGF (2 μg/ml), Hoechst 33342, 20 mM glucose, and 1% bovine serum albumin for 15 min at 37 °C. Images were captured using an EVOS M5000 Imaging System (Thermo Fisher Scientific). Fluorescence intensity of pHrodo-EGF per cell was calculated using MetaMorph NX software (Molecular Devices).

### Analysis of EGFR signaling pathways

A549 cells were incubated with NE (300 mU/ml) in serum-free DMEM for 20 min at 37 °C in the presence or the absence of NE inhibitor (100 μg/ml). The medium was then replaced, and cells were stimulated with EGF (50 ng/ml) for 10 min, as previously described. The expression of pEGFR, AKT, and pAKT was determined by Western blotting using antibodies against pEGFR (catalog no.: 3777; Cell Signaling Technology), AKT (catalog no.: 9272; Cell Signaling Technology), and pAKT (catalog no.: 9271; Cell Signaling Technology).

### Wound healing assay

Wound healing assay was performed according to previously published protocols, with some modifications (51). Briefly, A549 cells were cultured in 24-well plates to form a confluent monolayer. Pipette tips (1000 μl) were used to scratch a wound on the midline of culture wells. The medium was changed to DMEM, and cells were treated with NE (300 mU/ml) in the presence or the absence of NE inhibitor (100 μg/ml) for 30 min at 37 °C. After 24 h of culture in DMEM supplemented with 10% FBS or EGF (50 ng/ml), cell proliferation was evaluated by measuring the difference in the area of wounds using a microscope (BIOREVO BZ-9000; Keyence) and ImageJ software (Media Cybernetics, Inc).

### Pneumococcal pneumonia model of mice

In this study, 9-week-old male BALB/c mice were infected with *S. pneumoniae* as previously described, with some modifications (52). Mice were anesthetized with a mixture of medetomidine hydrochloride, midazolam, butorphanol, and *S. pneumoniae* strain D39 (2.5 × 10^8^ colony-forming unit in 50 μl PBS) intratracheally using a MicroSprayer Aerolizer. The NE inhibitor, sivelestat sodium hydrate (100 mg/kg), was intraperitoneally administered to the NE inhibitor–administered group (n = 5) immediately after infection and three times every 6 h after infection. The amount of NE inhibitor used was the same as that used clinically for treating acute lung injury. Uninfected (n = 5) and infected mice (n = 5) were intraperitoneally injected with PBS. The BALF and lung tissues were collected 20 h after infection. EGFR levels in the BALF were determined by Western blotting using full-length antibody against EGFR (catalog no.: 54359; Cell Signaling Technology) and anti-EGFR intracellular domain antibody (catalog no.: ab52894; Abcam). EGFR expression in the lung tissue was analyzed by Western blotting using anti-EGFR antibody (catalog no.: ab52894; Abcam). All mice were maintained under standard conditions, according to the guidelines of our institution. All animal experiments were approved by the Animal Care and Use Committee of Niigata University (no. SA00002).

### Immunohistochemical analysis

The lungs of mice were obtained 20 h after infection with *S. pneumoniae*. Immunofluorescence analysis was performed as previously described (52), with some modifications. The lungs were fixed in 4% paraformaldehyde phosphate buffer solution (FUJIFILM Wako Pure Chemical Corporation) for 24 h, embedded in OCT compound (Sakura Finetek Japan) and frozen in liquid nitrogen. Sections were cut at 8 μm and mounted on glass slides. The sections were fixed in paraformaldehyde for 10 min and washed with PBS containing 0.1% Tween-20, followed by washing with PBS containing 0.1% Triton X-100, and then PBS alone. Sections were stained using rabbit monoclonal antibody against EGFR (catalog no.: 54359; Cell Signaling Technology) or rabbit polyclonal antibody against Ki67 (catalog no.: 28074-1-AP; Proteintech Group, Inc), followed by incubation with Alexa Fluor 488–conjugated goat anti-rabbit immunoglobulin G (catalog no.: A-11034; Thermo Fisher Scientific) and mounting with a cover glass using VECTASHIELD Mounting Medium DAPI (Vector Laboratories). Fluorescent images were captured using a confocal laser scanning microscope (Carl Zeiss). Fluorescence intensity of EGFR per cell was calculated using MetaMorph NX software.

### Western blotting

Total protein was extracted from cells using M-PER Mammalian protein extraction reagent (Thermo Fisher Scientific) supplemented with 1% Halt Protease and Phosphatase Inhibitor Cocktail. T-PER Tissue Protein Extraction Reagent (Thermo Fisher Scientific) was used for protein extraction from the lung tissue. Proteins (20 μg) were separated by standard SDS-PAGE using 7.5% or 12% acrylamide gels (Bio-Rad) and transferred to polyvinylidene difluoride membranes (Merck Millipore) by electroblotting. The membranes were incubated in blocking buffer (Starting Block; Thermo Fisher Scientific), followed by probing with primary antibodies. Specific bands were visualized using horseradish peroxidase–conjugated secondary antibody (catalog no.: 7074; Cell Signaling Technology) and chemiluminescence using ECL Select reagent (Cytiva). β-actin was used as a loading control, and an anti-β-actin antibody (catalog no.: 5125) was purchased from Cell Signaling Technology. Images were captured using ImageQuant LAS 4000 mini (Cytiva).

### Statistics

Data were analyzed using GraphPad Prism software, version 6.05 (GraphPad Software, Inc). All results are presented as the mean ± SD. Group means were compared using one-way ANOVA with Tukey’s multiple comparison tests. *p* Values of 0.05 or less were considered statistically significant.

## Data availability

All data described are contained within the article.

## Supporting information

This article contains supporting information.

## Conflict of interest

The authors declare that they have no conflict of interest with the contents of this article.
