# Supplementary material for: Degradation of EGFR on lung epithelial cells by neutrophil elastase contributes to the aggravation of pneumococcal pneumonia
Source: J Biol Chem. 2023 Apr 27;299(6):104760. doi: 10.1016/j.jbc.2023.104760 (PMC10220274; doi:10.1016/j.jbc.2023.104760)
Supplement: Supporting Figures S1–S6 [file mmc1.pdf]

SUPPORTING INFORMATION

Degradation of EGFR on lung epithelial cells by neutrophil elastase contributes to the aggravation of pneumococcal pneumonia

Toshihito Isono, Satoru Hirayama, Hisanori Domon, Tomoki Maekawa, Hikaru Tamura, Takumi Hiyoshi, Kridtapat Sirisereephap, Shoji Takenaka, Yuichiro Noiri, Yutaka Terao

Supplemental figures S1-S6

Figure S1

(A)

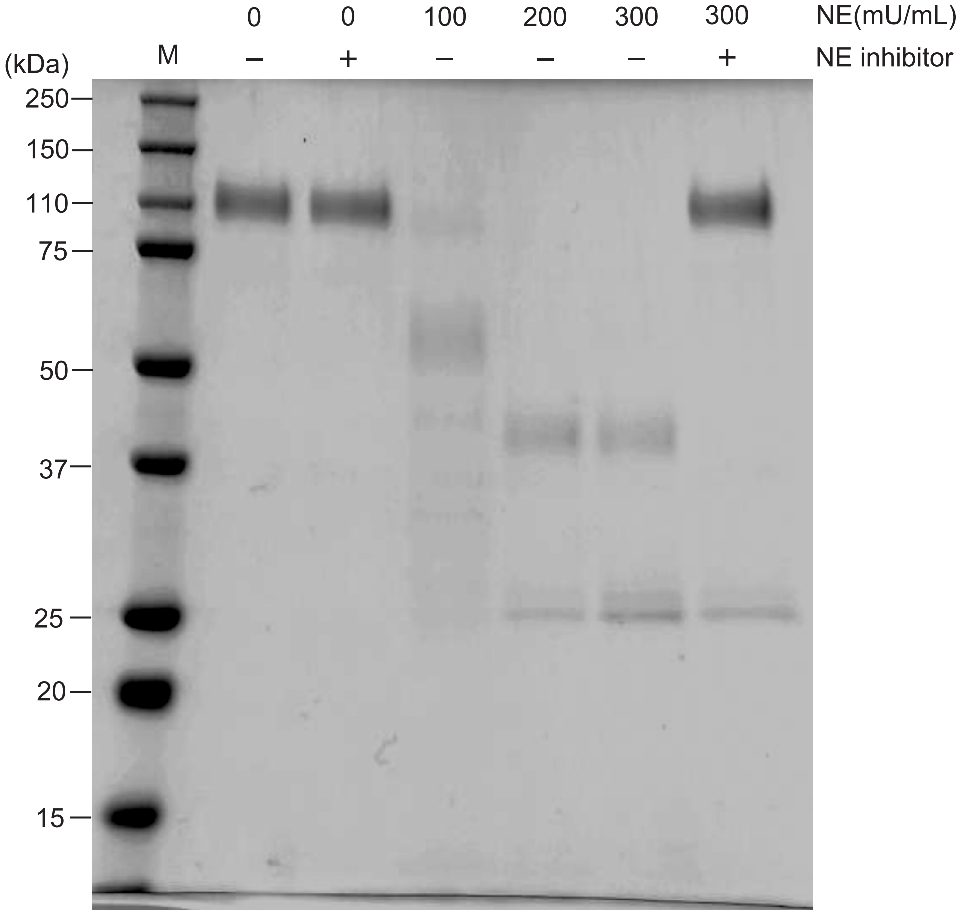

(B)

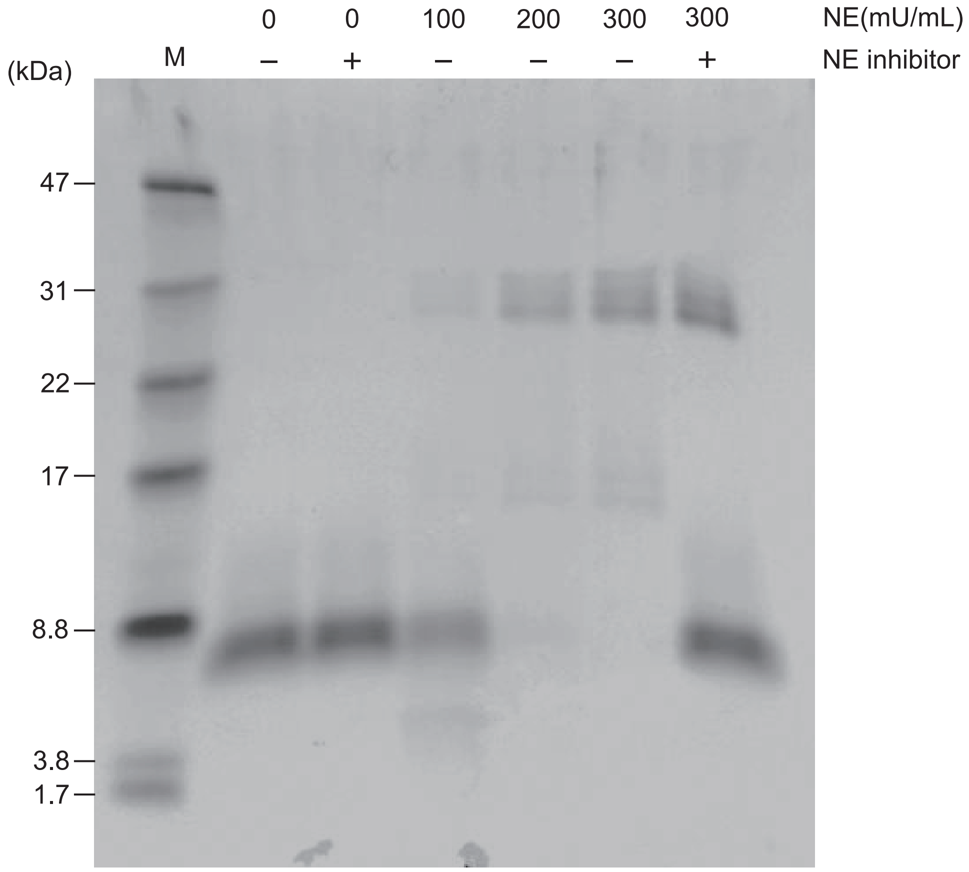

Figure S1 Original images of cleavage of rEGFR and rEGF by NE.

Unprocessed images shown in Figure 1.

Figure S2

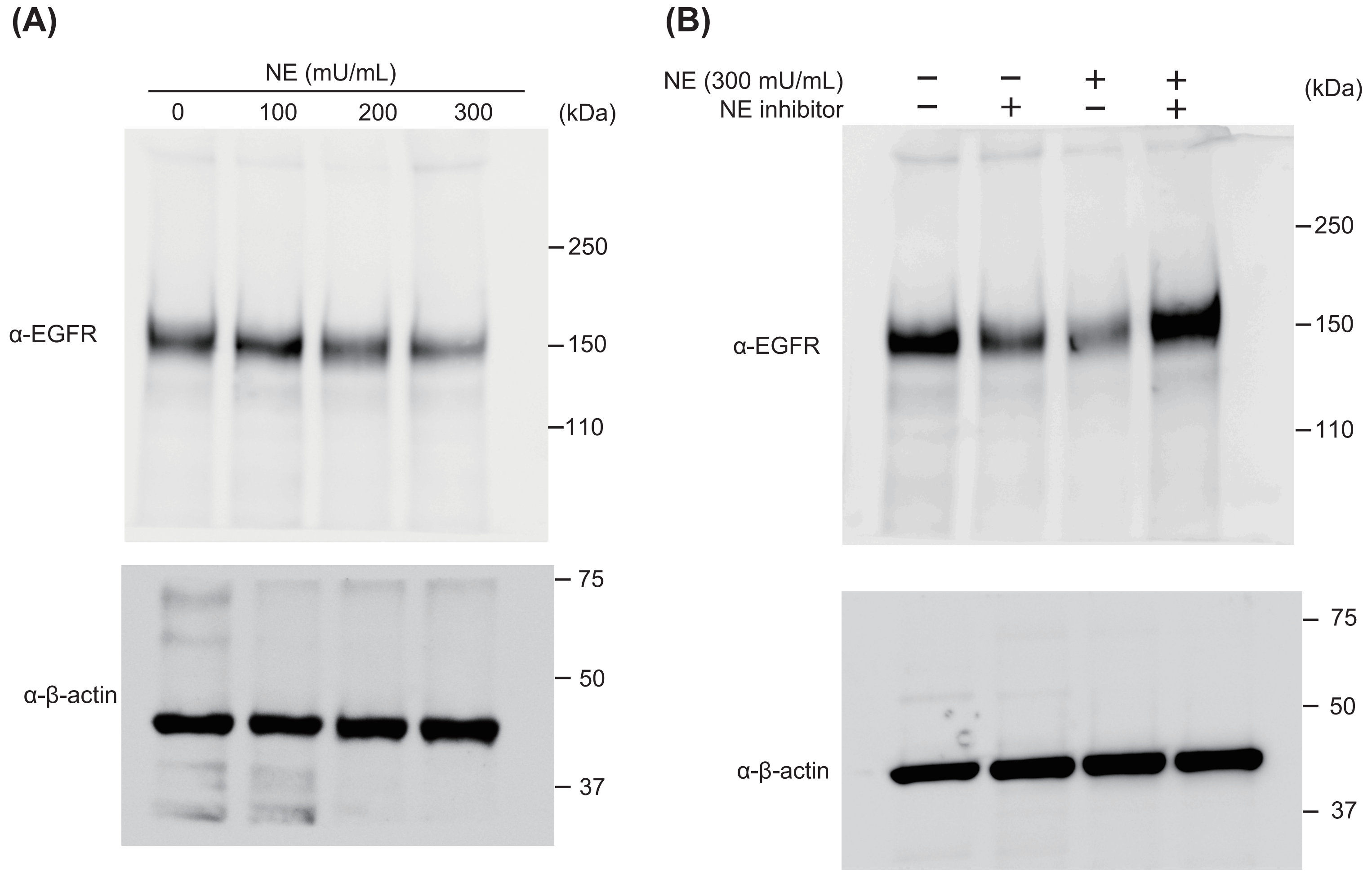

Figure S2 Original images of cleavage of EGFR on the surface of A549 cell by NE.

Unprocessed images shown in Figure 2.

Figure S3

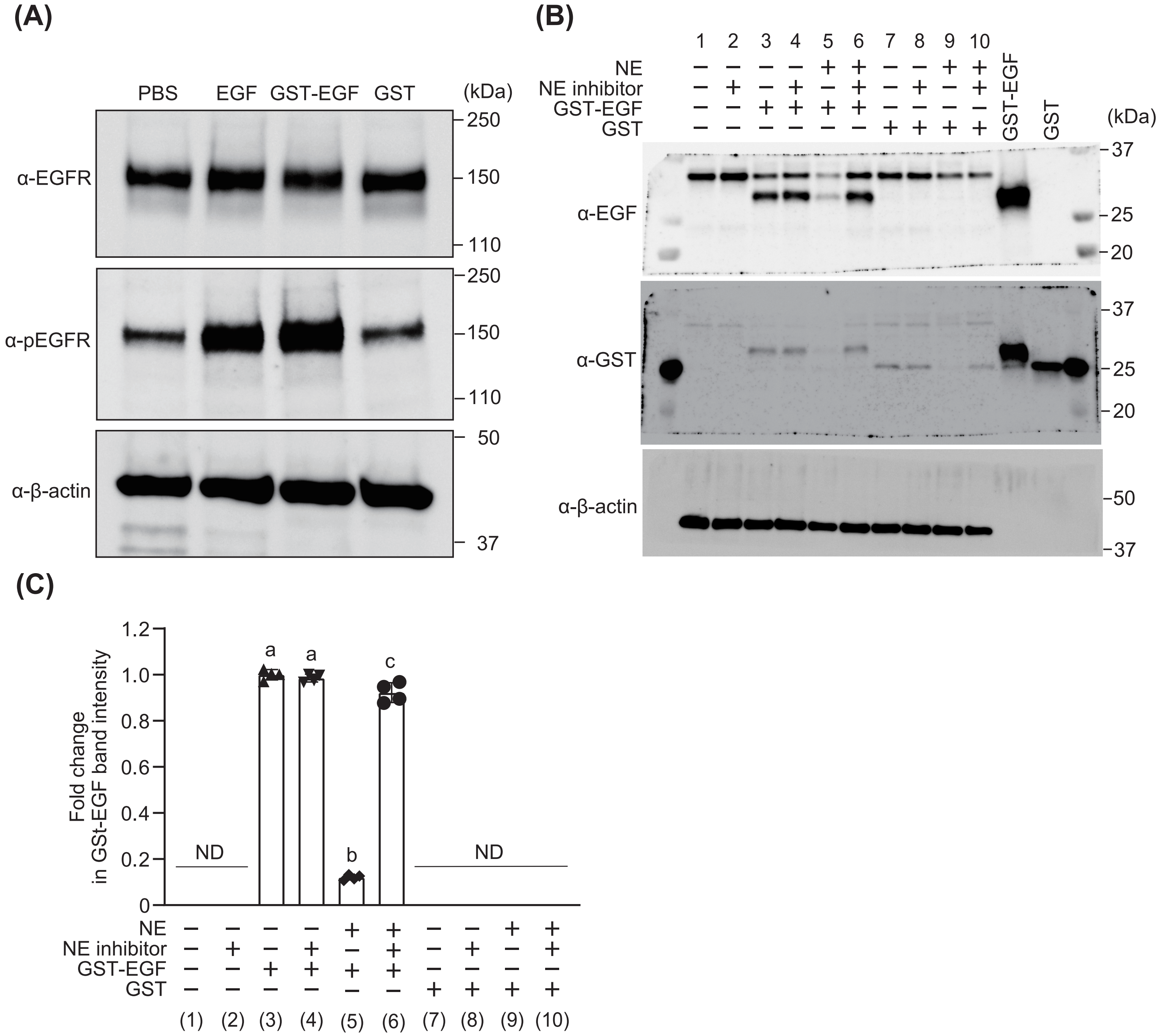

Figure S3 NE decreases the internalization of GST-EGF to EGFR.

(A) A549 cells were stimulated with EGF, GST-EGF, GST, and vehicle control (PBS) for 10 min. Then, the expression of pEGFR was determined by western blotting. (B) Unprocessed images of Figure 3A. (C) The intensity of GST-EGF band in Figure 3A detected by western blotting using anti GST antibody was quantified by the image analysis software. Unless otherwise indicated, no significant differences were observed between groups. Differences in letters between bars (a, b) indicate statistically significant differences between groups ($p < 0.05$). (3) vs (4): $p = 0.9571$, (3) vs (5): $p < 0.0001$, (3) vs (6): $p < 0.0001$, (4) vs (5): $p < 0.0001$, (4) vs (6): $p = 0.0003$, (5) vs (6): $p <0.0001$

Figure S4

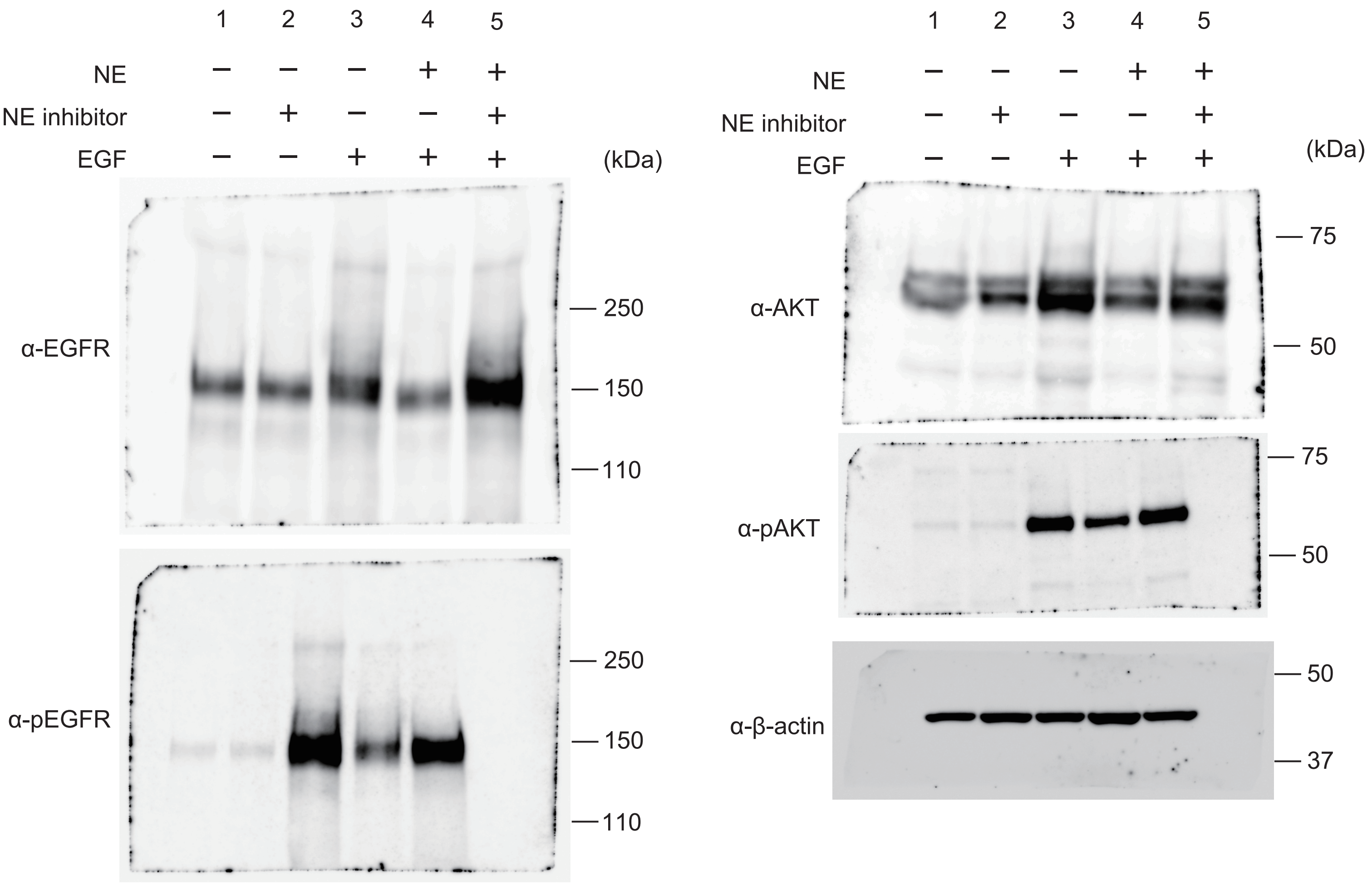

Figure S4 Original images of activation of EGFR in A549 cells treated with NE.

Unprocessed images shown in Figure 4.

Figure S5

(A)

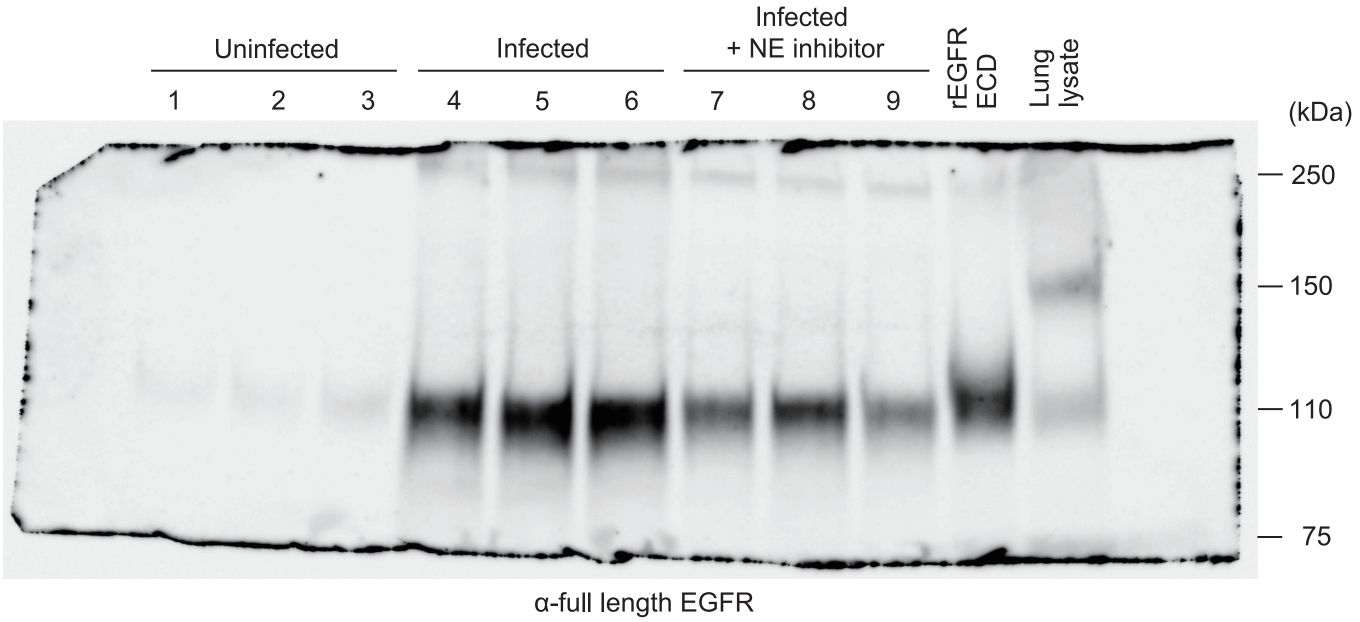

(B)

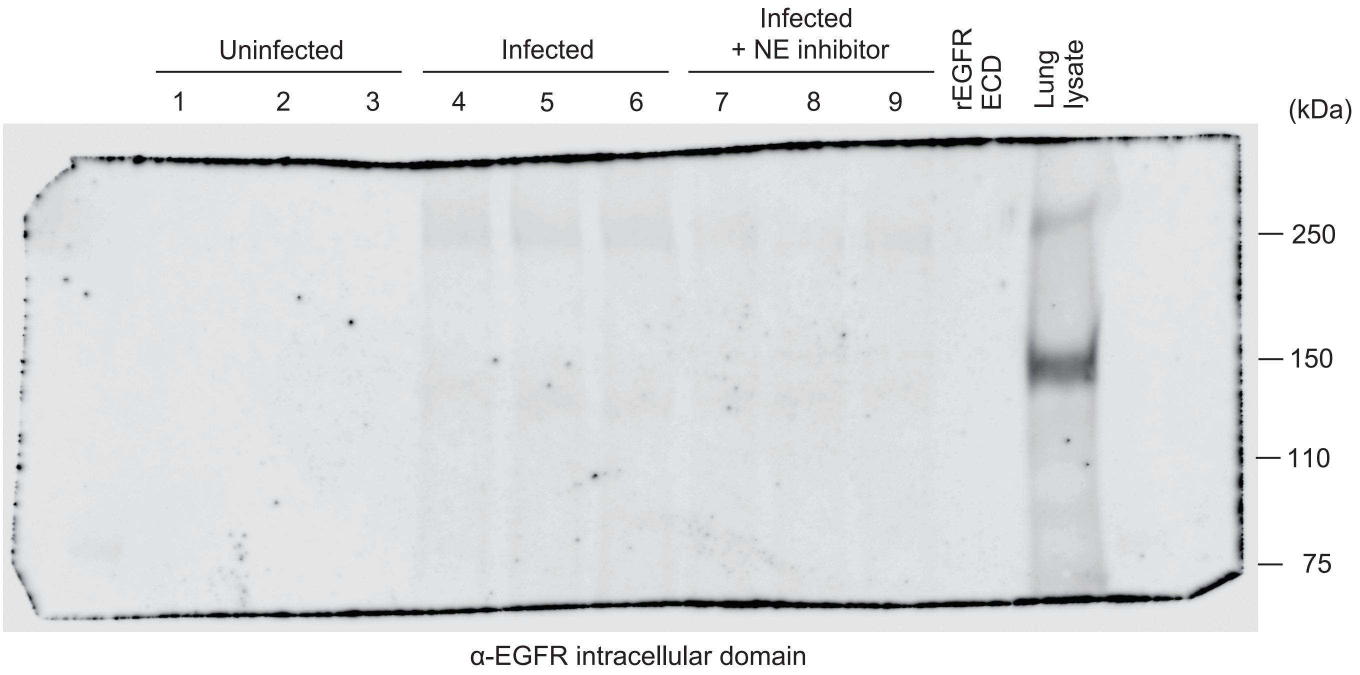

Figure S5 Original images of the detection of EGFR fragments in the BALF of mouse pneumococcal pneumonia model.

Unprocessed images shown in Figure 6.

Figure S6

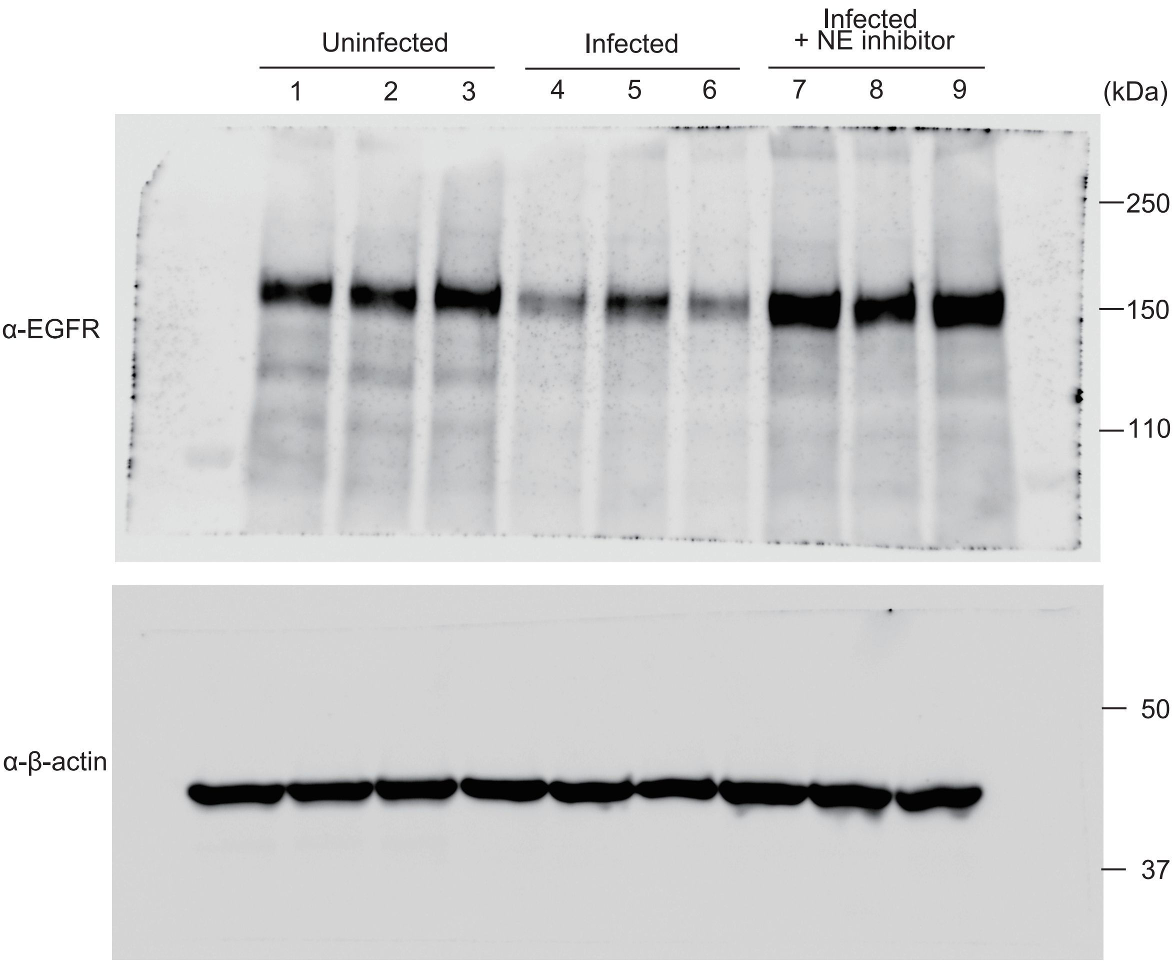

Figure S6 Original images of the expression of EGFR in lung tissue of mouse pneumococcal pneumonia model. Unprocessed images shown in Figure 8C.
